# Physical, psychological and nutritional outcomes in a cohort of Irish patients with metastatic peritoneal malignancy scheduled for cytoreductive surgery (CRS) and heated intrapertioneal chemotherapy (HIPEC): An exploratory pilot study

**DOI:** 10.1371/journal.pone.0242816

**Published:** 2020-12-09

**Authors:** Lisa Loughney, Noel McCaffrey, Claire M. Timon, Joshua Grundy, Andrew McCarren, Ronan Cahill, Niall Moyna, Jurgen Mulsow

**Affiliations:** 1 ExWell Medical, Dublin, Ireland; 2 School of Health and Human Performance, Dublin City University, Dublin, Ireland; 3 The Royal College of Surgeons in Ireland, Dublin, Ireland; 4 School of Nursing, Psychotherapy and Community Health, Dublin City University, Dublin, Ireland; 5 National Centre for Peritoneal Malignancy, Mater Misericordiae University Hospital Dublin, Dublin, Ireland; 6 Department of Computing, Dublin City University, Dublin, Ireland; 7 Department of Colorectal Surgery, Mater Misericordiae University Hospital, Dublin, Ireland; Royal Prince Alfred Hospital, AUSTRALIA

## Abstract

**Background:**

Treatment for peritoneal malignancy (PM) can include cytoreductive surgery (CRS) and heated intrapertioneal chemotherapy (HIPEC) and is associated with morbidity and mortality. Physical, psychological and nutritional outcomes are important pre-operatively. The aim of this pilot study was to investigate these outcomes in patients with PM before and after CRS-HIPEC.

**Methods:**

Between June 2018 and November 2019, participants were recruited to a single-centre study. Primary outcome was cardiopulmonary exercise testing (CPET) variables oxygen uptake (VO_2_) at anaerobic threshold (AT) and at peak. Secondary outcome measures were upper and lower body strength, health related quality of life (HRQoL) and the surgical fear questionnaire. Exploratory outcomes included body mass index, nutrient intake and post-operative outcome. All participants were asked to undertake assessments pre CRS-HIPEC and 12 weeks following the procedure.

**Results:**

Thirty-nine patients were screened, 38 were eligible and 16 were recruited. Ten female and 6 male, median (IQR) age 53 (42–63) years. Of the 16 patients recruited, 14 proceeded with CRS-HIPEC and 10 competed the follow up assessment at week 12. Pre-operative VO_2_ at AT and peak was 16.8 (13.7–18) ml.kg^-1^.min^-1^ and 22.2 (19.3–25.3) ml.kg^-1^.min^-1^, upper body strength was 25.9 (20.3–41.5) kg, lower body strength was 14 (10.4–20.3) sec, HRQoL (overall health status) was 72.5 (46.3–80) % whilst overall surgical fear was 39 (30.5–51). The VO_2_ at AT decreased significantly (p = 0.05) and HRQoL improved (p = 0.04) between pre and post- CRS-HIPEC. There were no significant differences for any of the other outcome measures.

**Conclusion:**

This pilot study showed a significant decrease in VO_2_ at AT and an improvement in overall HRQoL at the 12 week follow up. The findings will inform a larger study design to investigate a prehabilitation and rehabilitation cancer survivorship programme.

## Introduction

Peritoneal malignancy (PM) develops in a thin layer of tissue that lines the abdomen and is most commonly a result of metastatic spread of advanced primary tumours including colorectal, ovarian, appendix, gastric, and pancreatic. Treatment can include cytoreductive surgery (CRS) followed by heated intrapertioneal chemotherapy (HIPEC) and, in some cases, both neoadjuvant and adjuvant chemotherapy. CRS aims to remove the primary tumour, if present, and all sites of macroscopic peritoneal metastases whereas HIPEC targets residual microscopic disease. Although CRS-HIPEC can improve quality of life (QoL) and survival in selected patients [1–4], it is associated with extreme multisystem stress and carries a level of risk of morbidity and mortality [5–7].

Prehabilitation has been recently recommended by the Enhanced Recovery After Surgery (ERAS) Society [8] and the Peritoneal Malignancy Workshop [9] for people with PM scheduled for CRS-HIPEC. The evidence base on the effectiveness of prehabilitation which incorporates exercise, psychological and nutritional interventions is growing with encouraging outcomes in colorectal cancer surgery [10,11]. To date, however, no studies have reported prehabilitation interventions or indeed observed prehabilitation outcomes in this patient group although studies have explored QoL [12,13] and nutrition [14] as stand-alone outcomes.

Adequate pre‐operative physiological reserve is required to meet the functional demands of the surgical stress response, including increased cardiac output and delivery of oxygen [15,16]. Cardiopulmonary exercise testing (CPET) provides assessment of the integrative submaximal and maximal exercise responses involving the pulmonary, cardiovascular, muscular and cellular oxidative systems and provides an objective measure of physiological reserve required to withstand the stress of surgery [17]. To our knowledge, no study has reported CPET in this patient group however extensive research shows that low pre-operative fitness levels (CPET derived variable: oxygen uptake (VO2) at anaerobic threshold (AT) [18], is associated with increased post-operative morbidity and mortality in major surgical groups.

Psychological factors have an impact on surgical outcomes in both the short and long term including worse physiological surgical outcomes and post-operative QoL [19]. Surgical fear is commonly reported in the time before major surgery and can be linked to chronic pain, functional limitations and QoL, post-operatively [20]. QoL in people with PM scheduled for CRS-HIPEC has been measured using different tools and shown to return to baseline within 9–12 months following the procedure [12,13].

Poor nutrition prior to CRS-HIPEC is associated with increased length of stay [14]. Assessment of nutritional status before and after major surgery, and in patients with cancer, is now recommended by the European Society for Enteral and Parenteral Nutrition (ESPEN) [21,22]. For patients undergoing surgery, the general guidance is the prevention and treatment of undernutrition (the correction of undernutrition before surgery and the maintenance of nutritional status after surgery). Energy and protein requirements can be estimated with 25–30 kcal/kg and 1.5 g/kg ideal body weight [21,22]. Anticancer treatment (e.g. chemotherapy) and tumour complications (e.g., mechanical bowel obstruction, perforation, fistulisation) can negatively impact the quality of dietary intake for cancer patients, by affecting overall nutritional status [23]. Moreover, weight loss as a result of inadequate dietary intake is associated with poor tolerance of treatment, poor treatment outcomes, morbidity, and mortality [24].

To our knowledge, no study has reported physical, psychological and nutritional outcomes combined in patients with PM scheduled for CRS-HIPEC. Therefore, the aim of this pilot study was to investigate physical, psychological and nutritional status in patients with PM before and after CRS-HIPEC.

## Materials and methods

### Study design

This exploratory pilot study was an observational cohort study in patients with PM scheduled for CRS-HIPEC. Research ethics was approved by the Research Ethics Committee at Dublin City University (DCU) (DCUREC/2017/133) and the Mater Misericordiae University Hospital (MMUH) (1/378/1962). Participants were fully informed of the experimental procedures and provided with a plain language statement before written informed consent was obtained in accordance with the Research Ethics Committee at DCU and the MMUH.

### Participants

Eligibility for inclusion included: age ≥18 years, peritoneal disease undergoing CRS-HIPEC; and WHO performance status ≤2. The exclusion criteria were; inability to provide informed consent, impaired cognitive function with a reduced ability to either understand advice /instructions from or communicate with the research team, declined surgery, pregnancy or breastfeeding, inability to perform cardiopulmonary exercise testing or exercise and absolute contraindications to exercise testing.

### Recruitment

**Fig 1:**
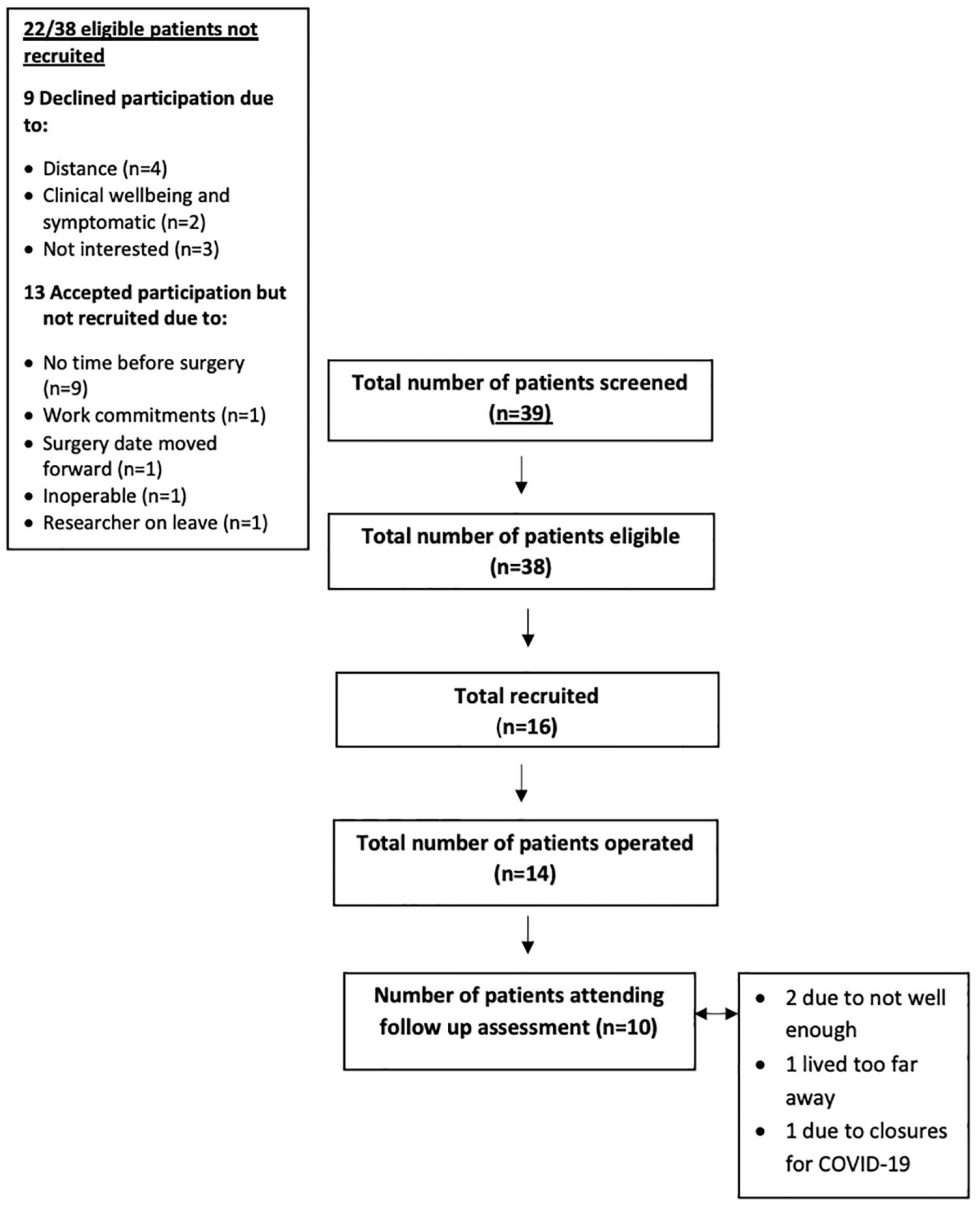
Study screening and recruitment algorithm.

The study screening and recruitment algorithm is summarized in Fig 1. From June 2018 to November 2019, eligible patients were given a patient information leaflet by either their surgical consultant or clinical nurse specialist in the outpatient cancer clinic at the MMUH. Interested patients were contacted by the research team (LL).

### Outcome measures

Outcome measures and time points of assessments are presented in S1 Table.

### Primary outcome

#### VO_2_ at AT and VO_2_peak

The primary outcome was VO_2_ at AT and VO_2_peak, measured using CPET. Participants were asked to refrain from caffeine and strenuous exercise 2 h prior to the test. All CPETs were performed in the School of Human Performance, Dublin City University, by the research team (LL, NMcC, NM). Every effort was made to coordinate research visits to coincide with clinical appointments (as patients travelled from different areas in Ireland to attend MMUH). Height and body mass were measured prior to the test using a stadiometer and electronic scale (model 707 balance scales: Seca GmbH, Hamburg, Germany), respectively. Body mass index (BMI) was calculated according to the World Health Organisation (WHO) classification: the weight in kilograms was divided by the square of the height in meters (kg/m^2^) [25]. WHO defines overweight as a BMI equal to or more than 25 kg/m^2^ and obesity as a BMI equal to or more than 30 kg/m^2^.

The CPETs were performed using an electromagnetically-braked cycle ergometer (Ergoline 2000, Rome, Italy) using a ramp protocol. Saddle height was measured and recorded at the first CPET and remained constant for the follow up CPET. Following a 2 min warm-up at 5–10 W, the incremental rise in work rate was pre-determined using the equation derived by Wasserman and colleagues [26]. The resistance was increased every minute using the pre-determined work-rate until the participant reached volitional fatigue. Participants were verbally encouraged to give their best effort. Breath by breath expired oxygen and carbon dioxide concentrations and ventilatory volumes were measured using open-circuit spirometry (COSMED K5, Rome, Italy). The system was calibrated according to the manufacturer’s procedures using a 3-L syringe.

The modified V-Slope method was used to identify the VO_2_ at AT [27]. Two physiological data assessors (LL and NM) undertook data interpretation, one blind to time point of assessment (NM). The multi-disciplinary team caring for the patients were not provided with any information regarding outcome measures.

A 12-lead ECG (Case 8000, Marquette GE, USA) was used to measure heart rate while pulse oximetry (Nonin 8500, Nonin Medical, INC, NH, USA) was used to measure oxygen saturation. Blood pressure was measured every 2 min using a sphygmomanometer and a stethoscope. Each participant received instructions on how to rate the Borg Scale of perceived exertion (Scale 0 to 10), which is a subjective rating of breathlessness for leg fatigue (assessed every 2 min during the test).

### Secondary outcomes

#### Strength

Hand grip strength was assessed on the dominant arm using a hydraulic handheld digital dynamometer (Takei 5401, Niigata, Japan) which consisted of gripping a handle with a strain-gauge and an analogue reading scale. The test was administered in a standing position with the upper arm held tight at the trunk and forearm at a right angle to upper arm. The gripping handle was adjusted to ensure the middle of their four fingers was resting on the handle. The handle was squeezed at maximum force and held for approximately 3 sec. Three trials separated ≥ 30 sec were performed and the average score was recorded in kg [28]. Normative data for hand grip strength for: males aged 40 years has been reported as mean (SD) 50.3 (10.3) kg, aged 50 years 47.6 (10.1) and aged 60 years 44.6 (9.2) whilst for: females aged 40 years 30.7 (6.3) kg, aged 50 years 28.7 (6.4), and aged 60 years 26.5 (6.2) [29].

Lower body strength was assessed using a 10 repetition sit to stand test from a seat height of 44–47 cm [30]. From a starting position with their arms crossed, feet placed flat on the floor pointing parallel to each other and approximately shoulder width apart, participants were instructed to stand up and sit down 10 times as fast as possible. Legs had to be fully extended and hips had to touch off the chair during the squat descent for the repetition to be valid. Participants were not allowed bounce from the chair when transferring from a seated to standing position. The time taken to complete 10 repetitions was measured using a stopwatch. Each participant performed two trials separated by 2 min, with the best score being recorded in sec.

#### Health related quality of life (HRQoL)

HRQoL was assessed using the EQ-5D questionnaire which is a simple descriptive profile of five health dimensions (mobility, self-care, usual activities, pain/discomfort and anxiety/depression) and a single index value for health status [31]. Participants were encouraged to fill out the questionnaire independently. Where assistance was required, a member of their family, their friend or one of the research team filled out the questionnaire for the participant.

#### Surgical fear

Surgical fear was assessed using the validated and reliable eight-item Surgical Fear Questionnaire [32]. The questionnaire consists of two subscales: fear of the short-term consequences of surgery and fear of the long-term consequences of surgery. All items were scored on an 11-point numeric rating scale ranging from 0 (not at all afraid) to 10 (very afraid). The range for short- and long-term fear is 0 (not at all afraid) to 40 (very afraid), and for the overall fear 0 (not at all afraid) to 80 (very afraid). Participants were encouraged to fill out the questionnaire independently. Where assistance was required, a member of their family, their friend or one of the research team filled out the questionnaire for the participant.

#### Nutritional intake

Nutritional intake was assessed using a researcher led web based 24 h recall tool, Foodbook24 (www.foodbook24.com). The validity and user evaluation of the tool are described elsewhere [33]. In brief, Foodbook24 facilitates the collection of dietary intake data from the previous 24 h period using a series of software prompts, probe questions and portion size images to ensure adequate capture of data and was calculated on reported food and beverage intake only, dietary supplements were not included. Traditionally the tool is used for the self-administered reporting of dietary intake data. However, in the present study a member of the research team (LL) assisted participants in completing the dietary recall using Foodbook24 at research visits. All data were energy adjusted. Nutrient intakes were adjusted for energy intake to control for confounding factors such as body weight, physical activity, metabolic efficiency etc. Macronutrients and their subtypes were adjusted for energy intake (% of energy).

### Post-operative outcome

Post-operative outcome was assessed by hospital length of stay, Clavien-Dindo classification and post-operative morbidity score (POMS) at days 3, 7 and 15 following surgery. The Clavien-Dindo classification of surgical complications consists of seven grades (Grade I, II, IIIa, IIIb, IVa, IVb and V) that rank post-operative complication severity (i.e. Grade I represents any deviation from the normal post-operative course without the need for pharmacological treatment or surgical, endoscopic and radiological interventions whilst Grade V represents death of a patient) [34]. The POMS is a validated 18-item tool that addresses nine domains of morbidity relevant to the post-surgical patient; pulmonary, infection, renal, gastrointestinal, cardiovascular, neurological, wound complications, haematological and pain [35]. For each domain, either the presence or absence of morbidity was recorded on the basis of precisely defined clinical criteria.

### Statistical analysis

Due to the nature of this novel pilot work, no priori formal power calculation was undertaken. Continuous variables are reported as median (IQR) and categorical variables as frequency (%). For continuous variables, a Wilcoxon test was used to compare pre- and post- CRS-HIPEC time points. Statistical significance was accepted at p ≤ 0.05. All analyses were performed with the statistical software IBM SPSS Statistics Ver.23 (IBM Corporation, Armonk, NY, USA).

## Results

Between June 2018 and November 2019, 38 out of 39 potential participants identified for screening met the inclusion criteria (Fig 1). Patient characteristics are presented in Table 1. Sixteen participants consented to participate in the study: 10 female and six male with a median age of 53 years (IQR 42–63). Two of the 16 patients recruited did not proceed with CRS-HIPEC. The median number of days between pre-operative CRS-HIPEC assessment and the procedure was 6 days (IQR 4–10).

**Table 1:** Participant characteristics.

	n = 16
Gender M:F (ratio)	6:10
Age (years) ^#^	53 (42–63)
Weight (kg) ^#^	76.8 (65.3–86)
BMI (kg/m^2^) ^#^	27.8 (25–31.8)
Comorbidity	
ASA Status^+^	
*2*	13 (81)
*3*	3 (19)
ECOG Performance Status^+^	
*0*	11 (69)
*1*	5 (31)
Past medical history^+^	
*Ischemic heart disease*	1 (6)
*Hypercholesterolemia*	2 (12)
*Diabetes (Type 2)*	1 (6)
*Chronic Obstructive Pulmonary Disorder*	0 (0)
Treatment Characteristics^+^	
*Colorectal with peritoneal metastatic disease*	6 (38)
*Appendix adenocarcinoma with peritoneal metastatic disease mets*	3 (19)
*Peritoneal mesothelioma*	1 (6)
*Pseudomyxoma peritonei*	5 (31)
*Teratoma*	1 (6)
PCI Score^+^	
*<10*	7 (44)
*10–20*	5 (31)
*>20*	4 (25)
CC Score^+^	
*0*	12 (75)
*1*	3 (19)
*2*	1 (6)
No. patients received cancer treatment^+^	
*Neoadjuvant cancer treatment*	5 (31)
*Adjuvant cancer treatment*	5 (31)
Duration of surgery (min) (n = 14) ^#^	377 (316.5–542.8)0)

Data are presented as

^**#**^median (IQR) and

^**+**^frequencies with percentage in parentheses.

Abbreviations: ECOG: Eastern Cooperation Oncology Group; ASA: American Society of Anaesthesiology; PCI: Peritoneal cancer index; CC: Completeness of cytoreduction.

### Pre-operative

#### Primary outcome

The VO_2_ at AT occurred at 16.8 (13.7–18) ml.kg^-1^.min^-1^ and VO_2_ peak at 22.2 (19.3–25.3) ml.kg^-1^.min^-1^, respectively (Table 2). All pre-operative CPET variables are presented in S2 Table.

**Table 2:** Pre-operative outcomes measures prior to CRS-HIPEC.

**Primary outcome**: CPET variables (n = 15)^#^		
VO_2_ at AT (l.min^-1^)	1.3 (1.1–1.4)	
VO_2_ at AT (ml.kg^-1^.min^-1^)	16.8 (13.7–18)	
VO_2_ at Peak (l.min^-1^)	1.7 (1.6–2.0)	
VO_2_ at Peak (ml.kg^-1^.min^-1^)	22.2 (19.3–25.3)	
**Secondary outcomes**		
**Strength**^#^		
*Upper body strength (kg)* (n = 14)	25.9 (20.3–41.5)	
*Lower body strength (sec)* (n = 16)	14 (10.4–20.3)	
**Health related quality of life: EQ5D questionnaire (n = 16)**^+^		
Mobility		
*No problems walking*	14 (87.5)	
*Slight problems walking*	2 (12.5)	
Usual Activities		
*No problems doing usual activities*	11 (69)	
*Slight problems doing usual activities*	3 (19)	
*Moderate problems doing usual activities*	0 (0)	
*Severe problems doing usual activities*	1 (6)	
*Unable to do usual activities*	1 (6)	
Self-care		
*No problems washing or dressing*	14 (87.5)	
*Slight problems washing or dressing*	2 (12.5)	
Pain / Discomfort		
*No pain or discomfort*	6 (37.5)	
*Slight pain or discomfort*	7 (44)	
*Moderate pain or discomfort*	2 (12.5)	
*Severe pain or discomfort*	1 (6)	
Anxiety / Depression		
*Not anxious or depressed*	7 (44)	
*Slightly anxious or depressed*	3 (18.5)	
*Moderately anxious or depressed*	6 (37.5)	
Health scale (between 0 and 100 best) ^#^	72.5 (46.3–80)	
**Surgical fear questionnaire (n = 16)**^#^		
*Short term fear*	22 (15–28.8)	
*Long term fear*	21.5 (12.3–26)	
*Overall fear*	39 (30.5–51	
**Exploratory outcomes**		
**Body mass index (n = 16)**^#^	27.8 (25–31.8)	
**Nutritional Intake (n = 15)**^§^		**IOM Daily Nutritional Goals**^1^
Energy (kcal/day)	1449.93 (1580.43)	1600–2000 kcal/day
Energy (MJ/ day)	6.07 (6.61)	
Protein (g/day)	55.57 (81.09)	
Percent Energy Protein^1^	15.31 (6.28)	10–35%
Carbohydrate (g/day)	163.18 (195.98)	
Percent Energy Carbohydrate^1^	42.82 (13.07)	45–65%
Total Sugars (g/day)	57.73 (88.53)	
Percent Energy Total Sugars^1^	16.48 (8.27)	<10%
Dietary Fibre (g/day)	11.66 (8.35)	22.4–28 g/day
Total Fat (g/day)	63.72 (60.26)	
Percent Energy Total Fat^1^	35.97 (10.39)	20–35%
Saturated fat (g/day)	28.95 (28.10)	
Percent Energy Saturated Fat^1^	14.74 (5.14)	<10%
Vitamin D (μg/10MJ)	2.65 (3.68)	15 μg/day
Vitamin B12 (μg/10MJ)	4.25 (6.18)	2.4 μg/day
Calcium (mg/10MJ)	907.04 (417.39)	1000–1200 mg/day
Iron (mg/10MJ)	13.29 (5.80)	8 mg/day
Sodium (mg/10MJ)	2629.06 (1599.58)	2300 mg/day

Data are presented as

^**#**^median (IQR),

^**+**^frequencies with percentage in parentheses and

^§^ mean (SD).

Abbreviations: CRS (Cytoreductive surgery)–HIPEC (heated intrapertioneal chemotherapy), VO_2_ at AT (oxygen uptake at anaerobic threshold), VO_2_ at Peak (oxygen uptake at peak exercise), (Institute of Medicine Dietary Reference Intakes).

^1^ Institute of Medicine, Nutritional Daily Goals for Males and Females Aged 51 Years+ (2006^37^, 2010^38^).

Note: Only 15/16 CPET data available due to system fault and dietary intake as error in data upload (internet connection); 14/16 upper body strength data as there was no testing equipment available to use.

For Foodbook24, all data were energy adjusted. Nutrient intakes were energy-adjusted, that is, the percentage of energy intake for macronutrients. Micronutrient intake is presented as intake as gram per milligram per milligram (g/mg/mg) per 10m MJ to account for total energy intake and to investigate the quality of the diet.

#### Secondary outcomes

Upper body strength was 25.9 (20.3–41.5) kg and the median (IQR) time to perform 10 repetitions of the sit to stand test measuring lower body strength was 14 (10.4–20.3) sec (Table 2). EQ5D questionnaire data for mobility, usual activities, self-care, pain/discomfort, and anxiety/depression is summarised in Table 2. The overall pre-operative health status was 72.5 (46.3–80) % (Table 2). The short term surgical fear was 22 (15–28.8), long term fear was 21.5 (12.3–26) and overall fear was 39 (30.5–51) (Table 2).

#### Exploratory outcomes

BMI was 27.8 (25–31.8) kg/m^2^ (Table 2). Nutritional intake is presented in Table 2.

### Post-operative

Of the 14 participants who underwent the CRS-HIPEC procedure, four were unable to attend a follow up appointment due to not being well enough (n = 2), living too far away (n = 1), and university closure/restrictions during the COVID-19 pandemic (n = 1). The median (IQR) number of weeks between CRS-HIPEC and follow-up assessment was 13 (12–14).

#### Primary outcome

**Fig 2:**
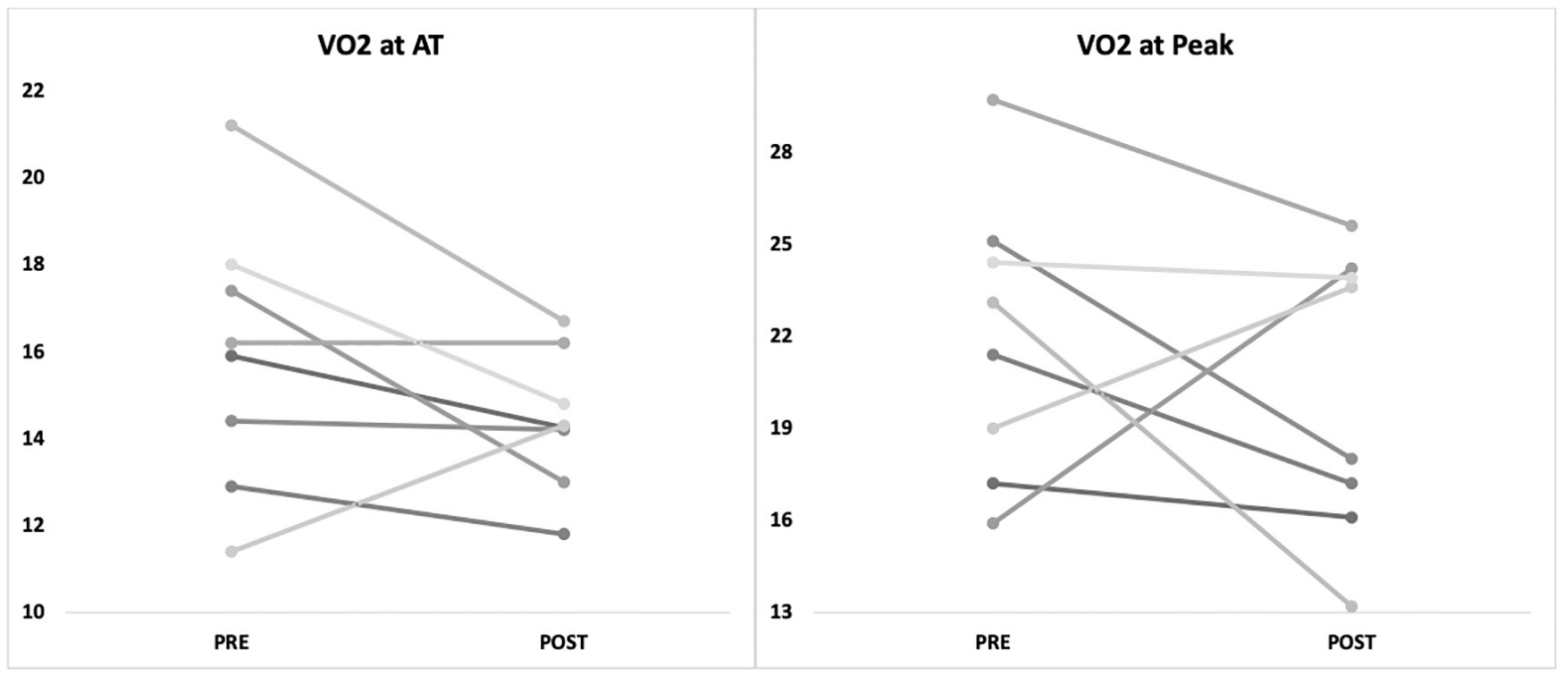
VO_2_ at AT and peak (ml.kg^-1^.min^-1^) pre- and post (at 12 weeks) CRS-HIPEC. Note: Lines link data-points (closed circles) for individual patients (n = 8).

Compared to pre-operative values, there was a reduction in VO_2_ at AT (p = 0.050) and no change in VO_2_peak post-operatively (Table 3). Individual changes from pre- to post- CRS-HIPEC for V̇O_2_ at AT are presented in Fig 2.

**Table 3:** Outcome measures pre- and post CRS-HIPEC.

Outcome measures	Pre CRS-HIPEC	Post CRS-HIPEC	P value
**Primary outcome**: CPET variables (n = 8) ^#^			
VO_2_ at AT (l.min^-1^)	1.2 (1.1–1.3)	1.1 (0.9–1.3)	0.050*
VO_2_ at AT (ml.kg^-1^.min^-1^)	15.9 (13.3–17.9)	14.3(12.3–15.2)	0.050*
VO_2_ at Peak (l.min^-1^)	1.7 (1.6–1.9)	1.6 (1.2–1.9)	0.116
VO_2_ at Peak (ml.kg^-1^.min^-1^)	20.9 (18.0–23.1)	20.8 (16.4–24.1)	0.499
**Secondary outcome**			
**Strength**^#^			
*Upper body strength (kg)* (n = 8)	26 (21.1–40.7)	25.2 (17–40.2)	0.069
*Lower body strength (sec)* (n = 10)	15.7 (9.8–24.5)	16.7 (9.7–18.7)	0.721
**Health related quality of life: EQ5D questionnaire (n = 10)** ^+^			
Mobility			0.317
*No problems walking*	8 (80)	9 (90)	
*Slight problems walking*	2 (20)	1 (10)	
Usual Activities			0.705
*No problems doing usual activities*	6 (60)	5 (50)	
*Slight problems doing usual activities*	3 (30)	5 (50)	
*Moderate problems doing usual activities*	0 (0)	0 (0)	
*Severe problems doing usual activities*	1 (10)	0 (0)	
Self-care			1.000
*No problems washing or dressing*	8 (80)	8 (80)	
*Slight problems washing or dressing*	2 (20)	2 (20)	
Pain / Discomfort			0.129
*No pain or discomfort*	4 (40)	6 (60)	
*Slight pain or discomfort*	4 (40)	4 (40)	
*Moderate pain or discomfort*	1 (10)	0 (0)	
*Severe pain or discomfort*	1 (10)	0 (0)	
Anxiety / Depression			0.024*
*Not anxious or depressed*	4 (40)	9 (90)	
*Slightly anxious or depressed*	2 (20)	1 (10)	
*Moderately anxious or depressed*	4 (40)	0 (0)	
Health scale (between 0 and 100 best) ^#^	70 (43.8–82.5)	80 (76.3–90)	0.041*
**Exploratory outcomes**			
Body mass index (n = 10) ^#^	28.9 (26.3–32.2)	28.3(26.1–30.9)	0.025*

Data are

^#^median (IQR) and

^**+**^frequencies with percentage in parentheses.

Note: Only 8/10 CPET data as system fault in pre-operative CPET (n = 1) and follow up CPET (n = 1).

Abbreviations: Abbreviations: CRS (Cytoreductive surgery)–HIPEC (heated intrapertioneal chemotherapy), VO_2_ at AT (oxygen uptake at anaerobic threshold), VO_2_ at Peak (oxygen uptake at peak exercise).

#### Secondary outcomes

From pre- to post- CRS-HIPEC, there was no change in upper body strength or lower body strength (Table 4) or for any of the HRQoL subscales (Table 4). There was a significant improvement (p = 0.04) however in HRQoL for overall health status.

**Table 4:** Nutrient intakes (Foodbook24) pre- and post- CRS-HIPEC.

	IOM Daily Nutritional Goals^1^	Pre CRS-HIPEC (n = 10)	Post CRS-HIPEC (n = 10)	P value
Energy (kcal/day)	1600–2000 kcal/day	1688.23 (1569.03)	1543.07 (472.09)	0.241
Energy (MJ/ day)		7.06 (6.56)	6.46 (1.98)	0.241
Protein (g/day)		57.77 (74.83)	75.39 (34.04)	0.445
Percent Energy Protein (%)	10–35%	16.52 (6.35)	15.70 (8.17)	0.721
Carbohydrate (g/day)		169.63 (186.92)	179.94 (51.92)	0.508
Percent Energy Carbohydrate (%)	45–65%	46.07 (14.41)	46.20 (10.93)	0.646
Total Sugars (g/day)		66.61 (106.43)	56.65 (50.41)	0.114
Percent Energy Total Sugars (%)	<10%	17.98 (6.66)	16.21 (9.93)	0.241
Dietary Fibre (g/day)	22.4–28 g/day	11.23 (10.43)	15.25 (9.24)	0.575
Total Fat (g/day)		70.36 (65.90)	60.38 (17.73)	0.285
Percent Energy Total Fat (%)	20–35%	32.88 (6.55)	37.82 (14.44)	0.721
Saturated fat (g/day)		32.70 (24.55)	24.05 (15.28)	0.333
Percent Energy Saturated Fat (%)	<10%	13.83 (6.67)	12.87 (8.32)	0.959
Vitamin D (μg/10MJ)	15 μg/day	2.25 (3.61)	3.08 (2.87)	0.646
Vitamin B12 (μg/10MJ)	2.4 μg/day	3.79 (5.78)	5.67 (6.28)	0.241
Calcium (mg/10MJ)	1000–1200 mg/day	939.28 (548.89)	999.13 (678.75)	0.241
Iron (mg/10MJ)	8 mg/day	13.10 (5.18)	13.37 (6.69)	0.074
Sodium (mg/10MJ)	2300 mg/day	2744.00 (1640.96)	2773.72 (1789.54)	0.508

Data are presented as mean (SD).

For Foodbook24, all data were energy adjusted. Nutrient intakes were energy-adjusted, that is, the percentage of energy intake for macronutrients. Micronutrient intake is presented as intake per gram per milligram per milligram (g/mg/mg) per 10 MJ energy intake to account for total energy intake and to investigate the quality of the diet.

Abbreviations: IOM (Institute of Medicine Dietary Reference Intakes).

^1^ Institute of Medicine, Nutritional Daily Goals for Males and Females Aged 51 Years+ (2006^37^, 2010^38^).

#### Exploratory outcomes

Compared to pre-operative values, there was a significant decrease (p = 0.025) in BMI (Table 4). Additionally, overall energy, protein and carbohydrate intake levels were low post-operatively. There was insufficient intake for dietary fibre, vitamin D and calcium, although the findings did not reach statistical significance (Table 4). Sufficient intakes of fat, iron and vitamin B12 intakes levels were observed. Intakes of sugar, saturated fat and sodium intake were in excess of recommended nutritional goals [36,37].

The median (IQR) length of hospital stay was 12 (10–17) days. Of the 14 participants that proceeded to CRS-HIPEC, eight (57%) participants experienced no complications, five (36%) participants experienced a Grade I complication and one (7%) participant experienced a Grade II complication. POMS data is reported descriptively in Table 5.

**Table 5:** Post-operative outcome data.

POMS Sub scales	Number of days post-surgery (n = 14)		
	*Day 3 (n = 14)*	*Day 7 (n = 14)*	*Day 15 (n = 4)*
*Pulmonary*	7 (50)	3 (21)	0 (0)
*Infectious*	9 (64)	9 (64)	3 (75)
*Renal*	11 (79)	3 (21)	0 (0)
*Gastrointestinal*	10 (71)	5 (36)	1 (25)
*Cardiovascular*	0 (0)	0 (0)	0 (0)
*Neurological*	0 (0)	0 (0)	0 (0)
*Hematological*	0 (0)	0 (0)	1 (25)
*Wound*	0 (0)	0 (0)	0 (0)
*Pain*	14 (100)	7 (50)	1 (25)

Data are presented as n (%) and median (IQR). The sub scales represent nine domains of post-operative morbidity.

Abbreviations: POMS (post-operative morbidity score).

## Discussion

To our knowledge, this is the first study to report physical, psychological and nutritional outcomes in patients with PM scheduled for CRS-HIPEC. This study demonstrates that participants had a reasonable level of physical fitness pre-operatively and that there was a significant reduction in physical fitness and improvement in overall HRQoL 12 weeks following the CRS-HIPEC procedure.

Pre-operative VO_2_ at AT in this study was 16.8 (13.7–18) ml.kg^-1^.min^-1^ which appears to be higher than other reported surgical groups where lower cut-points for VO_2_ at AT have been linked to poor post-operative outcomes. As a comparison, for patients undergoing hepatic transplant and resection, it was reported that a VO_2_ at AT < 9.9–11 ml.kg^-1^.min^-1^ was associated with an intensive care unit admission whilst 11 ml.kg^-1^.min^-1^was associated with 3 year survival. For patients undergoing pancreatic surgery, it was reported that a VO_2_ at AT of 10–10.1 ml.kg^-1^.min^-1^ was associated with increased hospital length of stay and morbidity and for patients undergoing intra-abdominal surgery, mortality was associated with a VO_2_ at AT of 10.9 ml.kg^-1^.min^-1^ and morbidity <10.1 ml.kg^-1^.min^-1^ [5]. It is important to note however that pre-operative evaluation for CRS-HIPEC includes a strict inclusion/exclusion criteria and involvement from a specialist multi-disciplinary team [38]. Although CRS has similar physiological insults to other surgeries, patients with PM are faced with the addition of thermal stress secondary to intraperitoneal administration of the heated chemotherapy agent [39], and therefore perhaps require a higher physiological reserve. The pre-operative physical fitness levels (VO2peak) of this patient group (median age) appear to be similar to their aged matched healthy counterparts [40], however given the wide variation in age it is difficult to confirm. Recovery has been described as the absence of symptoms and the ability of patients to perform activities as they could prior to the surgery [41]. Following CRS-HIPEC however, VO2 at AT significantly reduced by 10% overall. The overall decline in VO2 at AT may be important as 35% of participants in our study were starting/or scheduled to start adjuvant treatment at the time of follow up assessment and given the impact chemotherapy regimens have on physical fitness, participants in our study may have experienced a further decline in fitness in the months that followed. An additional follow up assessment at 6 –or 12 months would have allowed us to further investigate this.

Hand grip strength correlates well to post-operative outcome. Pre-operative upper body strength in this study was 25.9 kg. Specific to this patient group, no study has reported this which limits comparisons. However, one gastric cancer surgery study reported that 25 kg or less was associated with morbidity [42], whilst another oesophageal cancer study reported that a hand grip of less than 20 kg was associated with complications and mortality but greater than 40 kg was associated with better outcomes [43]. Following CRS-HIPEC, hand grip showed a decline of 0.8 kg which although non-significant may be important, as a recent systematic review reported 0.04 kg to 6.5 kg [44] as a clinically meaningful change. Hand grip strength pre and post- CRS-HIPEC appears to low when compared to aged matched counterparts (i.e. for healthy males and females aged 50 years mean (SD) 47.6 (10.1) kg and 28.7 (6.4), respectively) [29].

It has been previously reported that HRQoL following CRS-HIPEC (between 6–12 months) is the same as pre-operative HRQoL [12,13,45]. However, our study demonstrated that HRQoL significantly improved following CRS-HIPEC at the week 12 follow up. In other previous studies, HRQoL was measured using different tools such as Functional Assessment of Cancer Therapy (FACT), the European Organization for Research and Treatment of Cancer (EORTC) QLQ-C30, the Medical Outcomes Study Health Survey Short Form (SF-36), and the Eastern Cooperative Oncology Group (ECOG) performance status [12,13,45], which makes comparison of findings against our study difficult. It is interesting to note however that although HRQoL improved, physical fitness reduced at this time point in our study. A systematic review reported factors that negatively influence quality of life after CRS + HIPEC include higher age, gender (female), prolonged operation time, high completeness of cytoreduction (CC) score and peritoneal cancer index (PCI), adjuvant chemotherapy, post-operative complications, presence of a stoma, and disease recurrence within 12 months [13]. Although there were a higher number of female participants in our study (62.5%), 75% of the participants had a CC score of 0 and 44% had a PCI <10. Furthermore, 31% of the participants underwent adjuvant chemotherapy and 57% of participants experienced no complications. As there was a short-term follow up, we are unable to report disease recurrence at 12 months. Furthermore, both short- and long-term fear as well as overall fear of surgery in our study was on the mid-point of the scale, which is somewhat surprising given that surgery has been reported to be among one of the main fears people with cancer face [46]. Few studies have reported this measure in major surgical cancer groups making comparisons with our findings limited.

Guidance and the importance of nutrition in cancer care and surgery is highlighted in the ESPEN guidelines [21,22]. Poor diet quality has been shown to have negative implications for cancer recovery [47]. Findings from our study demonstrate that participants are maintaining adequate nutritional intake for the majority of dietary constituents however, potential nutritional deficits (dietary fibre, vitamin D and calcium) and high intakes of saturated fat were observed. This analysis may indicate suboptimal diet quality amongst this study population. The use of Foodbook24 in this study highlights the successful use of a web based technology to collect dietary intake data from patients with minimal cost, participant and researcher burden in a clinical setting. Future work in this area should consider combining dietary assessment with the subjective global assessment (SGA) for a more accurate assessment of nutritional status of cancer patients [48].

Prehabilitation supports people living with cancer to prepare for treatment by promoting healthy behaviours and prescribing exercise, psychological and nutritional interventions based on a person’s needs. A previous study reported that people with PM scheduled for CRS-HIPEC have a positive attitude towards prehabilitation [49]. Future work investigating cancer survivorship programmes including prehabilitation and rehabilitation in an adequately powered RCT in this patient group is required.

### Strengths and weaknesses of this study

The strength of this study is the novel approach to the assessment of patients with PM. CPET used a constant protocol and software; analysis was by two physiological assessors (one blind to time point of assessment), the multi-disciplinary team caring for the patients were not provided with any information regarding predictive measures (CPET variables).

Weaknesses include the nature of an exploratory pilot study including a small sample size. Although 38 patients were approached as being eligible for this study, 101 patients in MMUH underwent CRS-HIPEC during the study period which highlight missed opportunities to recruit. Of the 22/38 not recruited, 13 patients agreed to participate but nine of those were unable to due to the short timeline between initial contact with the patient and the surgical date. A dedicated health professional/researcher would have allowed more patients to be approached to participate in the study and possibly a higher recruitment rate. Furthermore, participants in this study had little comorbidity with a reasonable level of functioning, therefore it is likely they were a motivated group which may increase the risk of selection bias (impacting both internal and external validity). Heterogeneity exists due to the variation in patient age, tumour type and volume, extent of the surgery, post-operative complications and systemic chemotherapy. Although the recruitment sample was small, weaknesses also include the low number of participants who were unable to attend follow up assessment. A limitation to the dietary assessment was that the assessment was limited to a single day per time point. More frequent assessments at each time point would provide a more meaningful insight into overall diet quality, habitual dietary intake and contribution to overall nutritional status.

## Conclusion

This exploratory pilot study demonstrates firstly that participants in this study had a reasonable pre-operative physical fitness level. Secondly, that at 12 weeks following CRS-HIPEC, there was a significant reduction in physical fitness and improvement in overall HRQoL. Lessons learnt from this study will inform our future work as we propose to investigate a cancer survivorship programme including prehabilitation and rehabilitation to optimise patients physical, psychological and nutritional outcomes.

## Supporting information

S1 Table Outcome measures and time points of assessments. (DOCX)

S2 Table CPET variables pre CRS-HIPEC. (DOCX)

S3 Table CPET variables pre- and post CRS-HIPEC. (DOCX)
