# Supplementary material for: Physical, psychological and nutritional outcomes in a cohort of Irish patients with metastatic peritoneal malignancy scheduled for cytoreductive surgery (CRS) and heated intrapertioneal chemotherapy (HIPEC): An exploratory pilot study
Source: PLoS One. 2020 Dec 9;15(12):e0242816. doi: 10.1371/journal.pone.0242816 (PMC7725307; doi:10.1371/journal.pone.0242816)
Supplement: S1 Table — (DOCX) [file pone.0242816.s001.docx]

**S1 Table. Outcome Measures and Time Points of Assessments**

| **Outcome** | **Assessment measure** | **Baseline**  **(diagnosis)** | **Day 3 Post-surgery** | **Day 7 Post-surgery** | **Day 15 Post-surgery** | **Day 30 Post-surgery** | **12 Weeks Post-surgery** |
| --- | --- | --- | --- | --- | --- | --- | --- |
| **Primary endpoint** |  |  |  |  |  |  |  |
| Physical fitness | CPET | X |  |  |  |  | X |
| **Secondary endpoints** |  |  |  |  |  |  |  |
| Strength  *Upper body*  *Lower body* | Grip strength test  Sit-to-stand test | X  X |  |  |  |  | X  X |
| Health related quality of life | EQ-5D questionnaire | X |  |  |  |  | X |
| Surgical fear | Surgical fear questionnaire | X |  |  |  |  |  |
| **Exploratory endpoints** |  |  |  |  |  |  |  |
| Body mass index | Height/weight | X |  |  |  |  | X |
| Dietary Intake | Foodbook24 | X |  |  |  |  | X |
| Post-operative outcome | Post-operative morbidity score (POMS) |  | X | X | X | X |  |

Abbreviations: CPET – cardiopulmonary exercise test. “X” denotes measurement obtained at that time point
