# Supplementary material for: Physical, psychological and nutritional outcomes in a cohort of Irish patients with metastatic peritoneal malignancy scheduled for cytoreductive surgery (CRS) and heated intrapertioneal chemotherapy (HIPEC): An exploratory pilot study
Source: PLoS One. 2020 Dec 9;15(12):e0242816. doi: 10.1371/journal.pone.0242816 (PMC7725307; doi:10.1371/journal.pone.0242816)
Supplement: S2 Table — (DOCX) [file pone.0242816.s002.docx]

**S2 Table. CPET Variables Pre CRS-HIPEC**

| **CPET Variable**  **le** | **(n=15)** |
| --- | --- |
| VO_2_ at AT (l.min^-1^) | 1.3 (1.1 – 1.4) |
| VO_2_ at AT (ml.kg^-1^.min^-1^) | 16.8 (13.7 – 18) |
| VO_2_ at Peak (l.min^-1^) | 1.7 (1.6 – 2.0) |
| VO_2_ at Peak (ml.kg^-1^.min^-1^) | 22.2 (19.3 – 25.3) |
| WR at AT (W) | 82 (66 – 87) |
| WR at Peak (W) | 134 (114 – 152) |
| V_E_/VCO_2_ at AT | 27.6 (26.4 – 28.4) |
| V_E_/VCO_2_ at Peak | 31.1 (30 – 32.8) |
| PETCO_2_ at AT | 40.5 (38 – 42) |
| PETCO_2_ at Peak | 36 (35 – 38.3) |
| Breathing reserve | 32.8 (33.7 – 54.3) |

Data are median (IQR). Note: 15/16 CPET data due to system fault.

Abbreviations: VO_2_ at AT (oxygen uptake at anaerobic threshold), VO_2_ at Peak (oxygen uptake at peak exercise), WR at AT (work rate at anaerobic threshold), WR at Peak (work rate at peak exercise), V_E_/CO_2_ at AT (ventilatory equivalent for carbon dioxide at the anaerobic threshold), V_E_/CO_2_ at Peak (ventilatory equivalent for carbon dioxide at peak exercise), PETCO_2_ at AT (end tidal carbon dioxide at the anaerobic threshold), PETCO_2_ at Peak (end tidal carbon dioxide at peak exercise).
