# Supplementary material for: Physical, psychological and nutritional outcomes in a cohort of Irish patients with metastatic peritoneal malignancy scheduled for cytoreductive surgery (CRS) and heated intrapertioneal chemotherapy (HIPEC): An exploratory pilot study
Source: PLoS One. 2020 Dec 9;15(12):e0242816. doi: 10.1371/journal.pone.0242816 (PMC7725307; doi:10.1371/journal.pone.0242816)
Supplement: S3 Table — (DOCX) [file pone.0242816.s003.docx]

**S3 Table. CPET variables pre- and post CRS-HIPEC** (n=8)

| **CPET Variables** | **Pre CRS-HIPEC** | **Post CRS-HIPEC** | **P value** |
| --- | --- | --- | --- |
| VO_2_ at AT (ml.min^-1^) | 1.2 (1.1 – 1.3) | 1.1 (0.9 – 1.3) | 0.050* |
| VO_2_ at AT (ml.kg^-1^.min^-1^) | 15.9 (13.3 – 17.9) | 14.3 (12.3 – 15.9) | 0.116 |
| VO_2_ at Peak (ml.min^-1^) | 1.7 (1.6 – 1.9) | 1.6 (1.2 – 1.9) | 0.263 |
| VO_2_ at Peak (ml.kg^-1^.min^-1^) | 20.9 (18 – 23.1) | 20.8 (16.4 – 24.1) | 0.499 |
| WR at AT (W) | 86 (73 – 87) | 66 (45 – 75) | 0.018* |
| WR at Peak (W) | 134 (132 – 152) | 113 (98 – 140) | 0.028* |
| V_E_/VCO_2_ at AT | 27.9 (26.4 – 28.6) | 28.5 (25.7 – 31.7) | 0.463 |
| V_E_/VCO_2_ at Peak | 31.5 (30 – 33.3) | 31.5 (27.9 – 34.8) | 0.917 |
| PETCO_2_ at AT | 39.5 (38 – 41.3) | 40 (36.8 – 43.5) | 0.750 |
| PETCO_2_ at Peak | 35.5 (34.5 – 37.3) | 35.5 (32.8 – 40.5) | 0.496 |
| Breathing reserve | 44.5 (34.1 – 53.7) | 50 (39 – 61.2) | 0.173 |

Data are median (IQR). Note: 8/10 CPET data as system fault at baseline CPET (n=1) and follow-up CPET (n=1) limited paired comparison.

Abbreviations: VO_2_ at AT (oxygen uptake at anaerobic threshold), VO_2_ at Peak (oxygen uptake at peak exercise), WR at AT (work rate at anaerobic threshold), WR at Peak (work rate at peak exercise), V_E_/VCO_2_ at AT (ventilatory equivalent for carbon dioxide at the anaerobic threshold), V_E_/VCO_2_ at Peak (ventilatory equivalent for carbon dioxide at peak exercise), PETCO_2_ at AT (end tidal carbon dioxide at the anaerobic threshold), PETCO_2_ at Peak (end tidal carbon dioxide at peak exercise).
